# The Role of Alcohol Metabolism in the Pathology of Alcohol Hangover

**DOI:** 10.3390/jcm9113421

**Published:** 2020-10-25

**Authors:** Marlou Mackus, Aurora JAE van de Loo, Johan Garssen, Aletta D. Kraneveld, Andrew Scholey, Joris C. Verster

**Affiliations:** 1 Division of Pharmacology, Utrecht Institute for Pharmaceutical Sciences (UIPS), Utrecht University, 3584CG Utrecht, The Netherlands; marloumackus@gmail.com (M.M.); a.j.a.e.vandeloo@uu.nl (A.J.v.d.L.); j.garssen@uu.nl (J.G.); a.d.kraneveld@uu.nl (A.D.K.); 2 Institute for Risk Assessment Sciences (IRAS), Utrecht University, 3584CM Utrecht, The Netherlands; 3 Global Centre of Excellence Immunology, Nutricia Danone Research, 3584CT Utrecht, The Netherlands; 4 Centre for Human Psychopharmacology, Swinburne University, Melbourne, VIC 3122, Australia; andrew@scholeylab.com

**Keywords:** alcohol, hangover, ethanol, acetaldehyde, acetate, oxidative stress, malondialdehyde, 8-isoprostane

## Abstract

The limited number of available studies that examined the pathology of alcohol hangover focused on biomarkers of alcohol metabolism, oxidative stress and the inflammatory response to alcohol as potentially important determinants of hangover severity. The available literature on alcohol metabolism and oxidative stress is reviewed in this article. The current body of evidence suggests a direct relationship between blood ethanol concentration and hangover severity, whereas this association is not significant for acetaldehyde. The rate of alcohol metabolism seems to be an important determinant of hangover severity. That is, fast elimination of ethanol is associated with experiencing less severe hangovers. An explanation for this observation may be the fact that ethanol—in contrast to acetaldehyde—is capable of crossing the blood–brain barrier. With slower ethanol metabolism, more ethanol is able to reach the brain and elicit hangover symptoms. Hangover severity was also significantly associated with biomarkers of oxidative stress. More oxidative stress in the first hours after alcohol consumption was associated with less severe next-day hangovers (i.e., a significant negative correlation was found between hangover severity and malondialdehyde). On the contrary, more oxidative stress at a later stage after alcohol consumption was associated with having more severe next-day hangovers (i.e., a significant positive correlation was found between hangover severity and 8-isoprostane). In conclusion, assessment of biomarkers of alcohol metabolism suggests that fast elimination of ethanol is associated with experiencing less severe hangovers. More research is needed to further examine the complex interrelationship between alcohol metabolism, the role of acetaldehyde and oxidative stress and antioxidants, and the pathology of the alcohol hangover.

## 1. Introduction

The alcohol hangover refers to the combination of negative mental and physical symptoms which can be experienced after a single episode of alcohol consumption, starting when blood alcohol concentration (BAC) approaches zero [1,2]. An increasing body of the scientific literature is addressing the negative consequences of having hangovers for cognitive functioning including memory [3,4,5,6], and potentially endangering daily activities such as driving a car [7,8]. In contrast, research on the pathology of the alcohol hangover remains limited. Reviews on this topic suggest a variety of possible causes of the alcohol hangover [9,10,11], such as the role of the presence of ethanol and its metabolites in the blood, oxidative stress, and the immune response to alcohol consumption. However, the data presented in these reviews to support these hypotheses are limited. In this article, the current scientific evidence is reviewed and hypotheses are formulated on how alcohol metabolism and oxidative stress are related to the development of the alcohol hangover.

### 1.1. Alcohol Metabolism and Hangover Severity

The majority of ethanol is eliminated via oxidative processes in the liver [12,13,14]. Metabolism of ethanol is a two-step process, driven by the action of two enzymes, alcohol dehydrogenase (ADH), which oxidizes ethanol to acetaldehyde, and aldehyde dehydrogenase (ALDH), which oxidizes acetaldehyde to acetate. In both cases, the speed of both conversions is determined by the presence of the co-factor nicotinamide adenine dinucleotide (NAD^+^). A second pathway for alcohol breakdown, which is especially active in subjects who consume alcohol chronically, or in others after consuming large amounts of alcohol, is the microsomal ethanol oxidizing system (MEOS) [15]. This reaction is catalyzed by CYP2E1 and requires the co-factor nicotinamide adenine dinucleotide phosphate (NADP^+^), rather than NAD^+^, to convert ethanol into acetaldehyde. A third, relatively minor pathway involves the activity of catalase in liver peroxisomes in which ethanol functions as an electron donor for the reduction of hydrogen peroxide to water. Together, these oxidative pathways account for over 90% of alcohol elimination. The other 10% of ethanol is metabolized via non-oxidative pathways [16].

Irrespective of the pathway of alcohol metabolism, ethanol and acetaldehyde are the key compounds researched in relation to alcohol hangover. There is, however, ongoing debate about their role in the pathology of the alcohol hangover [9,10,11], and given the paucity of empirical data, theoretically both ethanol and acetaldehyde concentrations could have a direct influence on hangover severity. Many core hangover symptoms (e.g., headache, nausea, apathy, and concentration problems) likely involve central processes. While systemic processes clearly play a role in aspects of alcohol hangover, exposure of the brain to ethanol or its metabolites may ultimately determine the pathogenesis of alcohol hangover (symptoms). Given this, it is important to investigate the capability of peripheral ethanol and acetaldehyde to enter the brain and exert central effects, including a hangover.

Ethanol’s molecular structure allows it to freely cross the blood–brain barrier (BBB), and peripheral blood ethanol concentrations correlate significantly with brain ethanol concentrations [17]. This is not the case for acetaldehyde. Although original studies claimed that acetaldehyde can cross the BBB [18,19,20,21], these studies have been criticized for methodological shortcomings [22,23], and the current consensus is that acetaldehyde does not readily cross the BBB [23,24]. This is primarily due to the abundance of ALDH in the BBB which rapidly converts acetaldehyde into acetate and water before it can pass the membrane [23,25]. Theoretically small amounts of acetaldehyde may enter the brain via the circumventricular organs (CVOs), where no BBB exists [26]. However, under normal drinking circumstances, most acetaldehyde is metabolized systemically (i.e., before it reaches these areas), meaning negligible amounts of acetaldehyde will enter the brain via this pathway [27,28]. As a result, unlike ethanol and acetate, acetaldehyde is physiologically compartmentalized, with independent and different concentrations in the periphery and centrally [29,30,31]. As such, in theory, peripheral acetaldehyde should have no direct influence on hangover severity. However, it is important to note that acetaldehyde may also be produced centrally, via catalase from ethanol that entered the brain, where it can exert direct effects or be further metabolized and be involved in producing oxidative stress [32].

Only a few studies have reported correlations between hangover severity and acetaldehyde concentrations. This may be because of the fact that acetaldehyde is quickly converted into acetate and water, or because its blood concentration during the hangover state often falls below the detection limit [33,34]. This makes it difficult to accurately detect acetaldehyde.

Ylikahri et al. [35] examined the physiological correlates of a hangover in 23 healthy men. The morning following alcohol consumption (1.5 g/kg), no significant correlations were found between peak blood ethanol, acetaldehyde concentrations, and hangover severity. In a subsequent study, Ylikhari et al. [36] confirmed that blood acetaldehyde concentration was not significantly correlated with hangover severity. In a naturalistic study, Van de Loo et al. [37] found that urine ethanol concentrations assessed the morning following drinking were significantly lower in drinkers who reported no hangover compared to hangover-sensitive drinkers (*p* = 0.027). Overall hangover severity was positively and significantly associated with the amount of urine ethanol in those who reported having a hangover (r = 0.571, *p* = 0.013). Finally, using a mixed field and Internet methodology, Scholey et al. [38] found that hangover severity was significantly associated with the previous night’s breath alcohol concentration (r = 0.228, *p* = 0.019). Together, the majority of these studies suggested a relationship between ethanol concentration and hangover severity.

**Figure 1:**
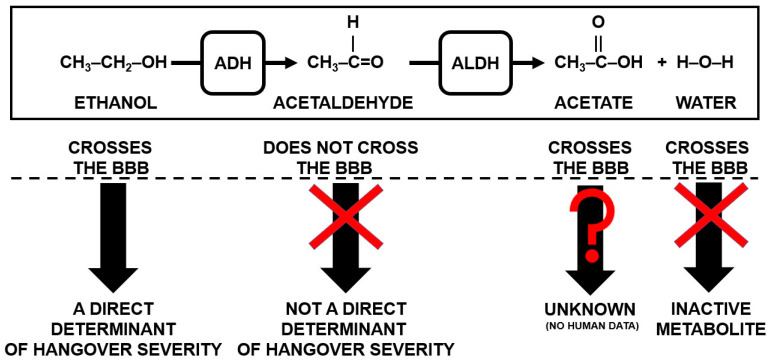
Alcohol metabolites and their hypothesized impact on the presence and severity of alcohol hangover. Abbreviations: BBB = blood brain barrier, ADH = alcohol dehydrogenase, ALDH = aldehyde dehydrogenase.

The end products of ethanol metabolism, acetate (acetic acid) and water, both readily cross the BBB [24,39]. Water is an ‘inactive’ metabolite of ethanol, while acetate has received very little research attention in the context of alcohol hangovers. One study assessed urine acetate and acetone levels during alcohol hangover and reported significantly increased concentrations [40]. Unfortunately, in the report of this study, no data were presented on the correlations of the concentrations of acetate and acetone with hangover severity. The findings from an animal study do suggest a possible relationship between blood acetate levels and hangover headache [41]. Using a headache model in rats, it was demonstrated that administering acetate contributed to the development of trigeminal pain in rats, whereas a similar correlation was not found for acetaldehyde. On the other hand, acetate is a common additive in food preservation [42] and is, for example, the main acid in vinegar. As such, acetate consumption as food constituent is considered safe and has not been associated with significant adverse effects when consumed at dietary levels. Taken together, the possible role of acetate in the pathology of the alcohol hangover deserves further investigation. Figure 1 summarizes the current hypothesis on how alcohol metabolism may be related to hangover severity.

As summarized in Figure 1, it is hypothesized that the concentration of peripheral ethanol may be the important correlate of hangover severity, as it can freely pass the BBB. In contrast, it is hypothesized that peripheral acetaldehyde does not play a direct role in the development of the alcohol hangover in the brain. However, as discussed elsewhere [43] and in this review, blood acetaldehyde concentrations are suggested to have indirect effects on hangover severity via oxidative stress or via the inflammatory response elicited by alcohol consumption.

### 1.2. Accelerating Ethanol or Acetaldehyde Breakdown in Reducing Hangover Severity

It has been hypothesized that accelerating alcohol metabolism will reduce hangover severity as ethanol and acetaldehyde are then quickly removed from the body. To further evaluate the literature on alcohol metabolism rate, it is important to take into account the fact that the alcohol breakdown rate is largely determined by the presence of two enzymes vital in catalyzing the alcohol metabolism, ADH and ALDH (see Figure 1), together with their co-factor NAD^+^.

**Figure 2:**
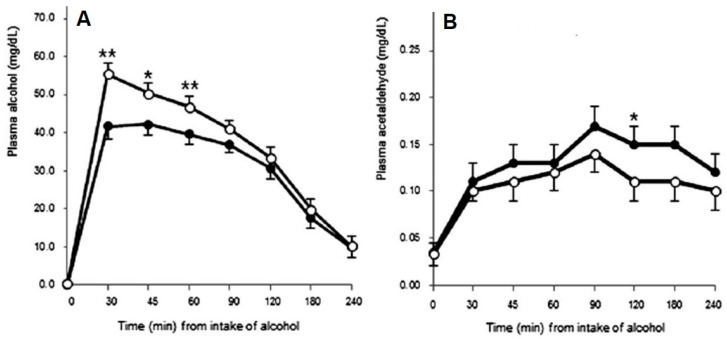
Effects of red ginseng on blood ethanol and acetaldehyde concentration. Alcohol and acetaldehyde levels over 240 min following alcohol intake in placebo (○) and test (●) with red ginseng groups. (**A**) Alcohol levels in plasma; (**B**) acetaldehyde levels in plasma. Values are expressed as means ± SEM. *p*-values are from nonparametric paired *t*-tests. * *p* < 0.05, ** *p* < 0.01. Reproduced from reference [46], with permission from the Royal Society of Chemistry.

There is evidence supporting the hypothesis that accelerating alcohol metabolism may reduce hangover symptom severity. For example, in Korea, red ginseng has been used for medical purposes for thousands of years, and research in rats and dogs revealed that red ginseng extract had short-term effects on ethanol metabolism in that it helped to reduce blood ethanol concentration [44,45]. Findings by Lee et al. [46] in human volunteers suggest that increasing the rate of alcohol metabolism may reduce hangover severity. Lee et al. [46] examined the effects of red ginseng on alcohol hangover severity. Higher blood ethanol concentrations (observed in the placebo condition vs. red ginseng condition at peak concentration, and first 60 min) were associated with significantly more severe hangovers (See Figure 2A). They further observed a rise in blood acetaldehyde levels 120 min after alcohol consumption, which was significantly greater after red ginseng than placebo (See Figure 2B). The reduction in ethanol and increase in acetaldehyde observed after red ginseng administration were followed by a significant reduction in next day hangover severity.

Dihydromyricetin (DHM), the active constituent of *Hovenia dulcis*, has been found to induce the expression of ethanol-metabolizing enzymes and reduce ethanol and acetaldehyde concentrations in rats [47]. Kim et al. [48] examined the effects of the fruit of *Hovenia dulcis* versus placebo on hangover severity in N = 26 healthy Asian men with heterozygous ALDH2. Blood samples were taken 1, 4 and 12 h after alcohol consumption and ethanol and acetaldehyde concentrations were determined. Hangover severity was assessed the next morning, 12 h after alcohol consumption. The authors reported a positive, significant relationship (r = 0.410, *p* = 0.003) between blood acetaldehyde concentration assessed 4 h after alcohol consumption and hangover severity. This finding suggests that higher acetaldehyde concentrations in the first hours after drinking are associated with having a more severe hangover the next day. However, critical further analysis of the same data by Van de Loo et al. [43] revealed a different outcome. Van de Loo et al. [43] examined data from the placebo condition of the study. It appeared that N = 10 subjects did not report a hangover and these were omitted from the analysis. Data from the remaining N = 16 subjects were analyzed, and it appeared that there were no significant correlations between blood acetaldehyde concentration assessed 1, 2 or 4 h after alcohol consumption and next-day hangover severity. In addition, no significant correlations were found between blood ethanol concentration and hangover severity. However, an indirect relationship between the presence of ethanol and hangover severity was found. The presence of ethanol was associated with increased levels of cytokines in the blood (interleukin (IL)-6 and tumor necrosis factor - alpha (TNF-α), of which the concentration was significantly and positively related to next-day hangover severity. This observation provides further evidence that a fast conversion of ethanol into acetaldehyde could be associated with experiencing less severe hangovers.

Finally, disulfiram is an ALDH2 inhibitor used in the treatment of alcoholism to prevent alcoholic patients from consuming alcohol. Its effects are established by inhibiting the breakdown of acetaldehyde into acetate and water, which results in accumulation of acetaldehyde. The effects of disulfiram start approximately 10 min after alcohol consumption and last for 1 h or more. Its effects include experiencing acute intoxication symptoms such as flushing, headache, nausea, vomiting, and sweating. Up to now, no studies have investigated the possible effects of disulfiram in the treatment of alcohol hangover.

Preclinical findings in animal studies and cell lines investigating natural compounds that enhance the activity of ADH and ALDH suggest that these products may possess properties which reduce or prevent alcohol hangovers [49,50]. In this context, recent research in social drinkers revealed that the dietary intake of two essential nutrients for the activation of ADH and ALDH was associated with hangover severity [51]. That is, there was a significant relationship between higher dietary intake of zinc and nicotinic acid and reporting less severe hangovers. Alternatively, acetaldehyde production can be influenced by modifying acetaldehyde producing microbiota [52]. High abundance of several microbiota such as *Rothia* is associated with increased production of acetaldehyde from ethanol [53]. A recent study [54] examined changes in the oral (saliva) microbiome of 15 young drinkers after an evening of heavy alcohol consumption. Compared to an alcohol-free day, the relative abundance of *Rothia*, *Streptococcus*, and *Veillonella* was significantly increased during the hangover state, whereas the relative abundance of *Prevotella*, *Fusobacterium*, *Campylobacter*, and *Leptotrichia* was significantly decreased. The largest change in saliva microbiome after heavy alcohol consumption was an increase in *Rothia*, which correlated significantly and negatively with reported hangover severity (r = −0.564, *p* = 0.036). Changes in other microbiota did not correlate significantly with hangover severity.

Genetic variety in ADH and ALDH also plays a role in alcohol metabolism [55], and this may affect the presence and severity of alcohol hangover. The ADH variants, of which ADH1B (subvariants *1, *2, and *3) and ADH1C (subvariants *1 and *2) are the most important in this context, differentially influence the breakdown rate of ethanol into acetaldehyde [56]. A relatively quick conversion from ethanol into acetaldehyde is observed in people possessing ADH1B*2, ADH1B*3 and ADH1C*1 alleles, whereas ethanol metabolism is relative slow in in people possessing the ADH1B*1 and ADH1C*2 allele [57]. The ADH1B*2 (common in people of Asian descent) and ADH1B*3 alleles (prevalent in people of African American decent) result in relative high blood concentrations of acetaldehyde (and thus lower blood ethanol concentrations) [58,59]. Additionally, subjects with an ALDH2*2 allele have a slower breakdown of acetaldehyde into acetate and water. Whereas acetaldehyde is usually quickly broken down, possession of the ALDH2*2 allele makes alcohol consumption unpleasant, because due to persistent elevated acetaldehyde concentrations in the blood, adverse effects such as flushing are frequently experienced [58]. Unfortunately, research into the genetics of alcohol hangover is limited. Two studies did report that subjects with Asian descent, possessing the ALDH2*2 allele, typically report significantly worse hangovers, and are more likely to experience hangovers at relatively lower alcohol consumption levels. [58,59]. Twin study by Slutske et al. [60] and Wu et al. [61] revealed that 45% [60] to 55% [61] of the frequency of experiencing hangovers. Forty-three percent of being hangover resistant could be explained by genetic variability [60]. These findings warrant further investigation into the impact of possessing different ADH and ALDH variants on the presence and severity of alcohol hangover.

**Figure 3:**
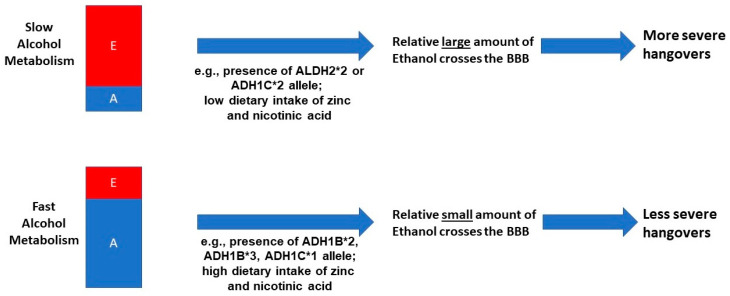
The impact of alcohol metabolism rate on hangover severity. Abbreviations: E = ethanol, A = acetaldehyde, BBB = blood brain barrier, ALDH = aldehyde dehydrogenase.

In conclusion, these studies support the hypothesis that a quick conversion of ethanol into acetaldehyde is associated with having less severe hangovers (see Figure 3). Contrary to popular belief, there is no published scientific evidence showing that any hangover product that claims to enhance the elimination of acetaldehyde is actually effective in reducing hangover severity. This, however, does not rule out the possibility that acetaldehyde may have indirect effects on the presence and severity of alcohol hangover. The fact that possessing the ALDH2*2 allele is associated with experiencing worse hangovers supports this possibility.

Up to now, one study directly associated alcohol elimination rate with hangover severity [62]. Data from N = 8 healthy volunteers who participated in both an acute study to investigate alcohol metabolism after alcohol consumption to achieve a BAC of 0.05% [63] and a naturalistic hangover study [64] were combined. In the acute alcohol study, breath alcohol content was assessed with a breathalyzer. Assessments were made every 5 min until subjects reached a BAC of zero. Using these data, the ethanol elimination rate was computed. These data were related to hangover severity reported in the hangover study, applying partial correlations correcting for estimated BAC. Hangover severity was assessed hourly from 09.30 to 15.30, using a one-item hangover severity scale ranging from 0 (absent) to 10 (severe) [65]. The analysis revealed significant negative correlations between hangover severity and ethanol elimination rate. In other words, those with a higher ethanol elimination rate (i.e., a faster conversion from ethanol into acetaldehyde) reported significantly lower hangover severity scores.

### 1.3. Oxidative Stress

**Figure 4:**
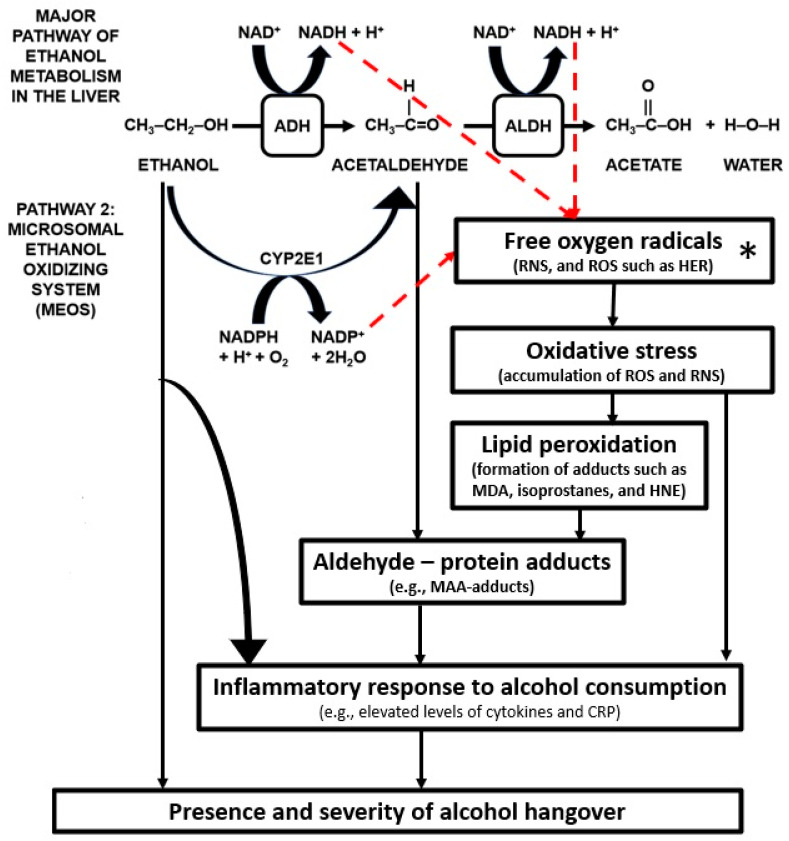
Oxidative stress and the inflammatory response to alcohol consumption. Arrows in red represent the relationship between alcohol metabolism and oxidative stress. Other arrows illustrate the pathways from oxidative stress to an inflammatory response and eliciting the alcohol hangover. Abbreviations: ADH = alcohol dehydrogenase, ALDH = aldehyde dehydrogenase, CRP = C-reactive protein, HER = hydroxyethyl radical, HNE = 4-hydroxy-2-nonenal, MAA = malondialdehyde – acetaldehyde, MDA = malondialdehyde (MDA), NAD^+^ = nicotinamide adenine dinucleotide, NADH = nicotinamide adenine dinucleotide (NAD) + hydrogen (H), NADP^+^ = nicotinamide adenine dinucleotide phosphate, NADPH = nicotinamide adenine dinucleotide phosphate + hydrogen (H), RNS = reactive nitrogen species, ROS = reactive oxygen species. Pathways contributing to ROS production are indicated by red dashed lines. * = free radicals are scavenged by antioxidants such as superoxide dismutase (SOD), catalase, and glutathione peroxidase, which may counteract lipid peroxidation and oxidative stress, and mitigate a subsequent inflammatory response.

Whereas in the liver the primary pathway of ethanol elimination is via ADH and ALDH activity, there exist alternative pathways for ethanol breakdown via the liver of which the microsomal ethanol oxidizing system (MEOS) is suggested to be the most important (See Figure 4).

The MEOS is more active after chronic alcohol use, as well as after drinking large quantities of alcohol. In addition, the MEOS is known to play a role in the link between alcohol metabolism and the inflammatory response consequent to alcohol consumption [43].

Ethanol is catalyzed by CYP2E1 into acetaldehyde, but in addition the MEOS also produces free (oxygen) radicals [66,67]. Free radicals are oxygen-containing molecules with unpaired electrons which allow them to easily react with other molecules. Free radicals, i.e., reactive oxygen species (ROS), are usually scavenged by antioxidants such as glutathione or superoxide dismutase (SOD). However, consuming large quantities of alcohol causes an imbalance between the amount of free radicals and antioxidants, i.e., excess levels of ROS such as hydroxyethyl radical (HER), called oxidative stress [66,67]. The abundance of free radicals elicits a process called lipid peroxidation, in which various byproducts of alcohol breakdown, called adducts, are formed [68,69]. Adducts are a combination of acetaldehyde or other aldehydes with a protein. Most notable in this regard are the aldehydes malondialdehyde (MDA) and 4-hydroxy-2-nonenal (HNE). Similar to acetaldehyde, these highly reactive aldehydes cannot pass through the blood brain barrier. In the presence of acetaldehyde, MDA can react with various proteins to form malondialdehyde–acetaldehyde adducts (MAA adducts). MAA adducts are known to have proinflammatory properties [70]. The MAA adducts are recognized by the body as foreign substances, and as a result an immune response is elicited [71,72,73], including increased secretion of cytokines and chemokines, as discussed elsewhere [43].

To counteract oxidative stress, various antioxidants (e.g., glutathione) and enzymes (e.g., catalase and superoxide dismutase) are produced endogenously. Cysteine is one of the precursors of glutathione, functioning as a semi-essential amino acid that binds acetaldehyde [74]. It has therefore been hypothesized that products increasing levels of antioxidants (e.g., glutathione, vitamin C, or vitamin E) may be effective as products to prevent hangovers [53]. However, most data to support these claims are indirect and comes from preclinical studies including animal research. For example, it has been shown that treatments that slowly release L-cysteine in the oral cavity significantly reduce saliva acetaldehyde concentration during alcohol consumption [75]. In rats, it was shown that a preparation of combined glutathione-enriched yeast (GEY) and rice embryo/soybean (RES) extracts significantly reduced both blood alcohol and acetaldehyde concentrations after an alcohol challenge [76]. Other research in rats revealed that administering electrolyzed-reduced water (ERW) was accompanied by activation of glutathione, and a significant increase in ADH and ALDH in the liver [77]. In the context of such preclinical findings, L-cysteine, its precursor N-acetyl-L-cysteine (NAC), and glutathione have been proposed as hangover treatments and are included as ingredients in various marketed hangover treatments. However, supportive peer-reviewed scientific data from clinical trials in humans on the efficacy of antioxidants in reducing or preventing hangovers are currently lacking [78,79,80,81]. Indeed, two recent double blind placebo controlled studies found no significant effect of a hangover treatment consisting of L-cysteine. Scholey et al. found no significant improvement of hangover severity after administering L-cysteine (650 mg) and B- and C-vitamins [82], and Eriksson et al. also did not demonstrate a significant reduction in hangover severity after administering L-cysteine (600 mg and 1200 mg) [83,84]. The reported data from a clinical trial on www.clinicaltrials.gov showed that NAC had no significant effects on reducing hangover severity but did produce more adverse effects than placebo (clinicaltrials.gov identifier NCT02541422). Another study examining NAC and hangover was terminated before completion (clinicaltrials.gov identifier NCT03104959), and the results from a third hangover study with glutathione are not reported (clinicaltrials.gov identifier NCT00127309).

To date, only one study has investigated both the biomarkers of oxidative stress and antioxidants in relationship to hangover severity [85]. Mammen et al. [85] examined the effect of a polyphenolic extract of clove buds (Clovinol) versus placebo on hangover severity in N = 16 healthy men, aged 25 to 55 years old. Blood ethanol and acetaldehyde concentration were determined before and at 0.5, 2, 4, and 12 h after alcohol consumption (240 mL of 42.8% McDowell’s V.S.O.P. Brandy; United Spirits Limited, Bangalore, India), and a hangover scale was completed 14 h after drinking. To assess oxidative stress, two biomarkers (8-isoprostane and malondialdehyde) were assessed, as well as two antioxidants, glutathione and superoxide dismutase (SOD). No significant difference in ethanol concentrations was found between placebo and Clovinol at any time point. After intake of alcohol alone (i.e., the placebo condition), biomarkers of oxidative stress increased over time, whereas antioxidant concentrations decreased. Mammen et al. [85] observed a significant reduction in hangover severity after Clovinol, which was associated with a significant reduction in acetaldehyde, 8-isoprostane and malondialdehyde concentration and a significant increase in glutathione and SOD. Van de Loo et al. [43] further evaluated the placebo data of the study by Mammen et al. This evaluation revealed no significant correlations between hangover severity and blood ethanol or acetaldehyde concentrations at any time point after alcohol consumption. In addition, concentrations of the antioxidants glutathione and SOD did not significantly correlate with hangover severity at any timepoint after alcohol consumption.

A significant negative correlation was found between hangover severity and blood malondialdehyde concentration at 0.5 h after alcohol consumption (r_B_ = −0.707, BCa 95%CI_B_ = −0.96, −0.16). Correlations between hangover severity and 8-isoprostane in the first hours after drinking were also negative, but did not reach statistical significance. At a later stage after drinking, a significant positive correlation was found between hangover severity and 8-isoprostane at 12 h (r_B_ = 0.475, BCa 95%CI_B_ = 0.06, 0.78), and the association between malondialdehyde at 12 h and hangover severity was also positive, but did not reach significance.

Together, these findings suggest that higher levels of oxidative stress in the first hours after alcohol consumption (i.e., quick metabolism of ethanol) are associated with less severe hangovers (a negative correlation was found), whereas high levels of oxidative stress and inflammation during hangover are associated with more severe hangovers (a positive correlation was found).

## 2. Conclusions

**Figure 5:**
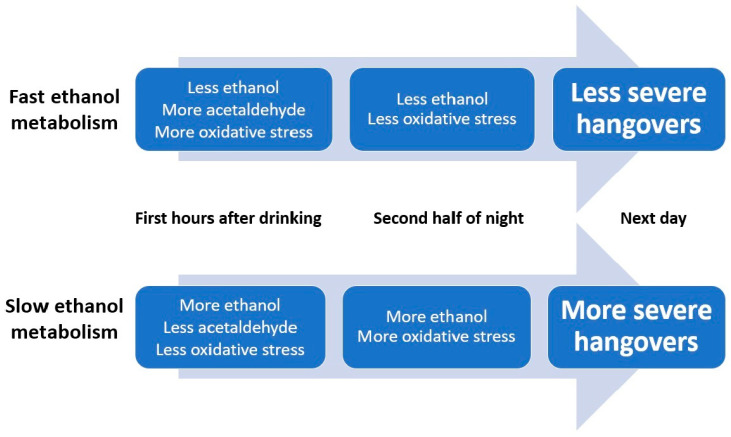
Schematic representation of the hypothesized relationship between alcohol metabolism and hangover severity.

The data summarized in this review suggest that the ethanol elimination rate is a critical determinant of hangover severity for a number of reasons. First, significant correlations have been found between ethanol concentration (but not acetaldehyde) and hangover severity. Second, nutrients, microbiota, and hangover treatments that speed up the conversion of ethanol into acetaldehyde are associated with less severe hangovers. Taken together, the data suggest that a more rapid conversion of ethanol to acetaldehyde and other aldehydes is associated with less severe hangovers. Based on the currently available data, the hypothesized relationship between alcohol metabolism and the presence and severity of alcohol hangover is schematized in Figure 5.

The association between blood ethanol content and hangover severity is physiologically plausible, because unlike acetaldehyde, ethanol can freely pass the BBB. Compared to fast metabolizers of alcohol, slow metabolizers will have relatively large amounts of circulating ethanol that can cross the BBB over a longer time period, increasing the relative probability of a more severe hangover.

Figure 5 also includes oxidative stress, suggesting that high levels of oxidative stress in the first hours after drinking are associated with less severe hangovers, whereas high levels of oxidative stress during hangover are associated with more severe hangovers. It should be taken into account, however, that at this moment limited data are available on the role of oxidative stress and antioxidants in the pathogenesis of alcohol hangover, and this observation was based on only one study [43,85]. Therefore, much more research attention is needed to confirm these findings and elucidate the exact relationship between oxidative stress and alcohol hangover. Additionally, more research is needed to investigate to what extent antioxidants can prevent or reduce alcohol hangover, oxidative stress and the inflammatory response to alcohol consumption are interrelated and may impact hangover severity. Finally, although no direct effects of acetaldehyde on hangover severity have been demonstrated, acetaldehyde does play a role in oxidative stress resulting in hangovers. It has also been shown that subjects with an ALDH2*2 allele report more severe hangovers than other drinkers. Therefore, more research is also needed in the role of acetaldehyde in the development of alcohol hangover. Notwithstanding the limited amount of research, it is evident that alcohol metabolism and ethanol elimination rate play a critical role in the presence and severity of the alcohol hangover.
